# Proliferating cell nuclear antigen expression in mucoepidermoid carcinoma of salivary glands

**DOI:** 10.1590/S1516-31802000000300004

**Published:** 2000-05-02

**Authors:** Weder Pereira Cardoso, Odilon Victor Porto Denardin, Abrão Rapoport, Vera Cavalcanti Araújo, Marcos Brasilino Carvalho

**Keywords:** Proliferating cell nuclear antigen, Mucoepidermoid Carcinoma, Salivary Gland, PCNA, Carcinoma mucoepidermóide, Glândulas salivares

## Abstract

**CONTEXT:**

Among the cytological and morphological properties of mucoepidermoid carcinoma, one of the most important criteria for measuring its biological behavior and aggressiveness is cell proliferation. In this way, immunohistochemical markers of cell proliferation have been found to be useful in tumor classification and have formed part of the prognostic and therapeutic studies of these pathologies.

**OBJECTIVE:**

To analyze 11 cases of mucoepidermoid carcinoma (MEC) using the proliferation activity marker (PCNA) and to determine its relationship to the grade of malignancy of these tumors.

**DESIGN:**

Correlation study.

**SETTING:**

Head and Neck Surgery Service of Heliópolis Hospital, São Paulo, Brazil.

**SAMPLE:**

Slides of 11 cases of primary mucoepidermoid carcinomas of salivary glands were prepared according to routine techniques employed in the Oral Pathology Department of the Dentistry Faculty of São Paulo University, Brazil. They were fixed in a 10% formaldehyde solution and stained with hematoxylin and eosin. After this preparation the tumors were classified as low, intermediate and high grade of malignancy, according to the criteria established by Seifert & Sobin and Auclair, Goode & Ellis. The slides were sent for immunohistochemical processing to evaluate the positivity of proliferating cell nuclear antigen using the streptavidin biotin technique.

**MAIN MEASUREMENT:**

The correlation between proliferating cell nuclear antigen expression and the histological malignancy grade in mucoepidermoid carcinoma of salivary glands.

**RESULTS:**

there were 4 cases (36%) of low grade, 4 cases (36%) of intermediate grade and 3 cases (27%) of high grade of malignancy. After a comparative study between histological features and immunohistochemical analysis, significant differences were observed (P < 0.01) for low, intermediate and high grades: 16.04%, 26.98% and 56.98% of proliferating cell nuclear antigen expression in mucoepidermoid carcinoma, respectively.

**CONCLUSION:**

The proliferating cell nuclear antigen expression increases with the grade of malignancy in mucoepidermoid carcinoma of salivary glands.

## resumo

**CONTEXTO:**

As características citológicas e morfológicas do Carcinoma Mucoepidermóide, um dos critérios mais importantes na aferição do seu comprometimento biológico e agressividade é a proliferação celular. Daí, a utilização do uso de marcadores imunohistoquímicos da proliferação celular na classificação dos tumores e na determinação do prognóstico e no estudo terapêutico dessas patologias.

**OBJETIVO:**

Analisar 11 casos de carcinoma mucoepidermóide (CME) através do antígeno nuclear de proliferação celular (PCNA) e determinar sua relação com o grau de malignidade destes tumores.

**TIPO DE ESTUDO:**

Estudo de correlação.

**LOCAL:**

Serviço de Cirurgia de Cabeça e Pescoço do Hospital Heliópolis, São Paulo, Brazil.

**AMOSTRA:**

As lâminas de 11 casos foram preparadas de acordo com a rotina técnica do Departamento de Patologia Oral da Faculdade de Odontologia da Universidade de São Paulo, correspondendo ao carcinoma mucoepidermóide primário de glândulas salivares, fixadas em solução de formaldeído a 10% e coradas com hematoxilina e eosina. Após o preparo, as neoplasias foram classificadas em baixo, intermediário e alto grau de malignidade, de acordo com o critério de Seifert & Sobin e Auclair & Ellis. As lâminas foram encaminhadas a estudo imunohistoquímico para avaliar a positividade do PCNA pela técnica de streptavidina.

**VARIÁVEIS ESTUDADAS:**

Correlação entre a expressão PCNA e o grau histológico de malignidade dos CME de glândulas salivares

**RESULTADOS:**

Haviam 4 casos (36%) de baixo grau, 4 casos (36%) de grau intermediário e 3 casos (27%) de alto grau de malignidade. Após o estudo comparativo entre os achados histológicos e a análise imunohistoquímica, diferenças significativas foram observadas (P<0,01) para os 3 graus de malignidade – 16,04%, 26,98% e 56,98% respectivamente – da expressão do PCNA no CME.

**CONCLUSÃO:**

A expressão do PCNA aumenta com o grau de malignidade do CME nas glândulas salivares.

## INTRODUCTION

The large number of histological types of tumors in salivary glands makes them a very heterogeneous group of neoplasias. The greatest difficulties in their identification are due to the morphological variety and complexity that compromise the histopathological diagnosis and classification of these tumors.^1^

Mucoepidermoid carcinoma (MEC) represents 5 to 12% of salivary gland tumors, with the most common site being the parotid gland (70 to 90%).^2–7^ In the oral cavity, the palate is the most affected site and the lip is the site with least incidence.^8^

In addition to the cytological and morphological properties of these neoplasias, one of the most important criteria for the measurement of their biological behavior and aggressiveness is cell proliferation. In this way, immunohistochemical markers of cell proliferation have been found to be useful in tumor classification and have formed part of the prognostic and therapeutic studies of these pathologies.^9,10^

The proliferating cell nuclear antigen (PCNA) identified by Myiach et al^11^ is a 36 kd, acidic, nonhistone nuclear protein that helps the delta DNA polymerase in DNA synthesis.^12^ This protein has a high concentration in the late G1 and early S phases, diminishes in the G2 phase and is almost absent in the M phase.^12,13^

The evaluation of cell proliferation using PCNA is comparable to and, under certain conditions, superior to the traditional methods of mitotic figure count using optical microscopy, tritiated timidin uptake and flow cytometry.^9,14–16^

This study was performed to verify the presence of a correlation between PCNA expression and the histological malignancy grade in MEC of salivary glands.

## METHODS

In a retrospective analysis of the records of the Head and Neck Surgery Service of Heliópolis Hospital, 25 cases of MEC of salivary glands were found between 1978 and 1997. In eleven cases the sample blocks were suitable for reevaluation of the histopathological diagnosis and for immunohistochemical analysis.

The clinical data on the patients (age, sex, race, presence of symptoms and outcome) and tumors (site and size) were collated.

### Diagnostic tests

**Figure 1:**
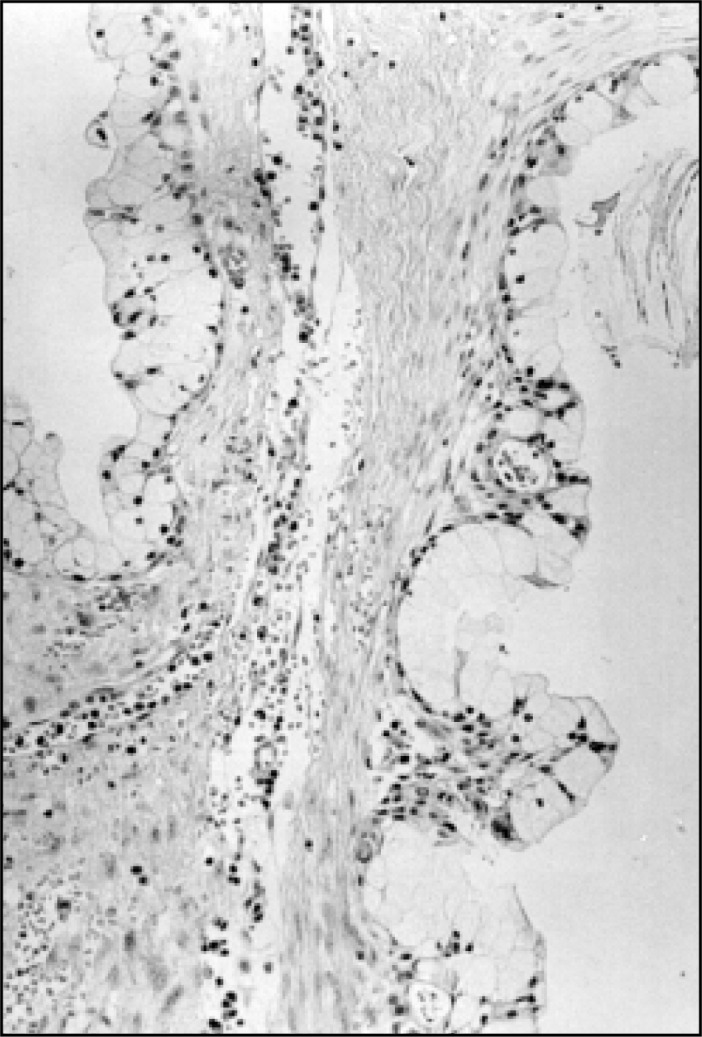
Low malignancy grade MEC of a salivary gland. Note the mucin cell with wide, clear cytoplasm and the nucleus displaced to the periphery, lining the cystic spaces. (Stained with hematoxylin and eosin with magnification of 250x).

**Figure 2:**
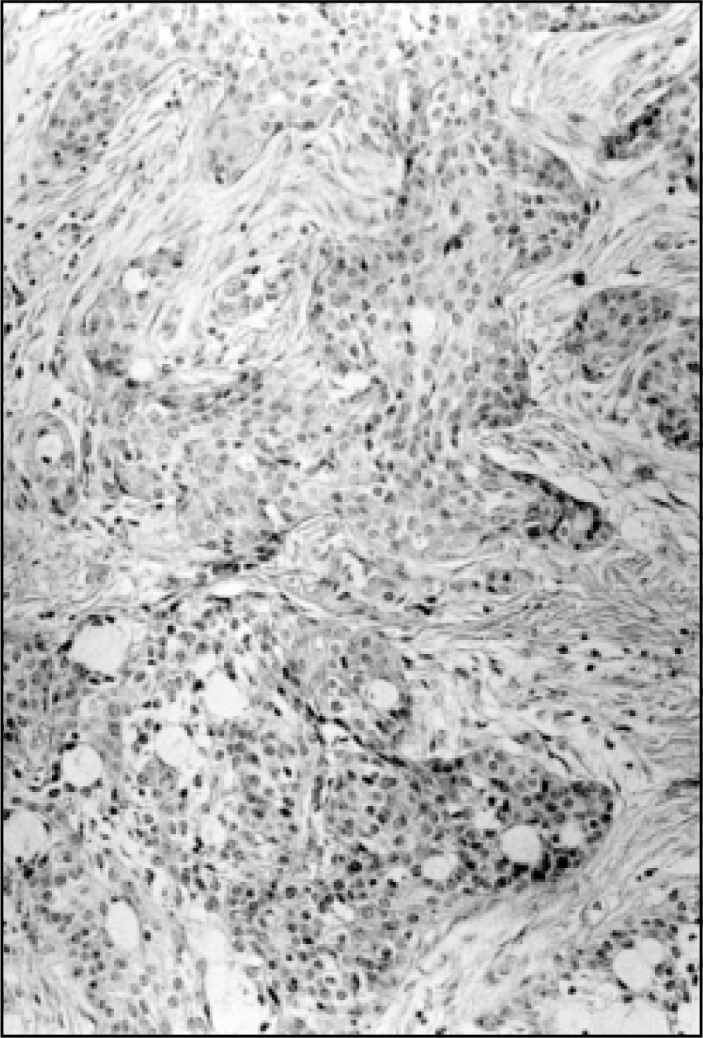
Intermediate malignancy grade MEC of a salivary gland. Note the proliferation of small epidermoid cells with eosinophilic cytoplasm and ovoid nucleus and connective tissue stroma. Few cystic spaces are shown. (Stained with hematoxylin and eosin with magnification of 250x).

**Figure 3:**
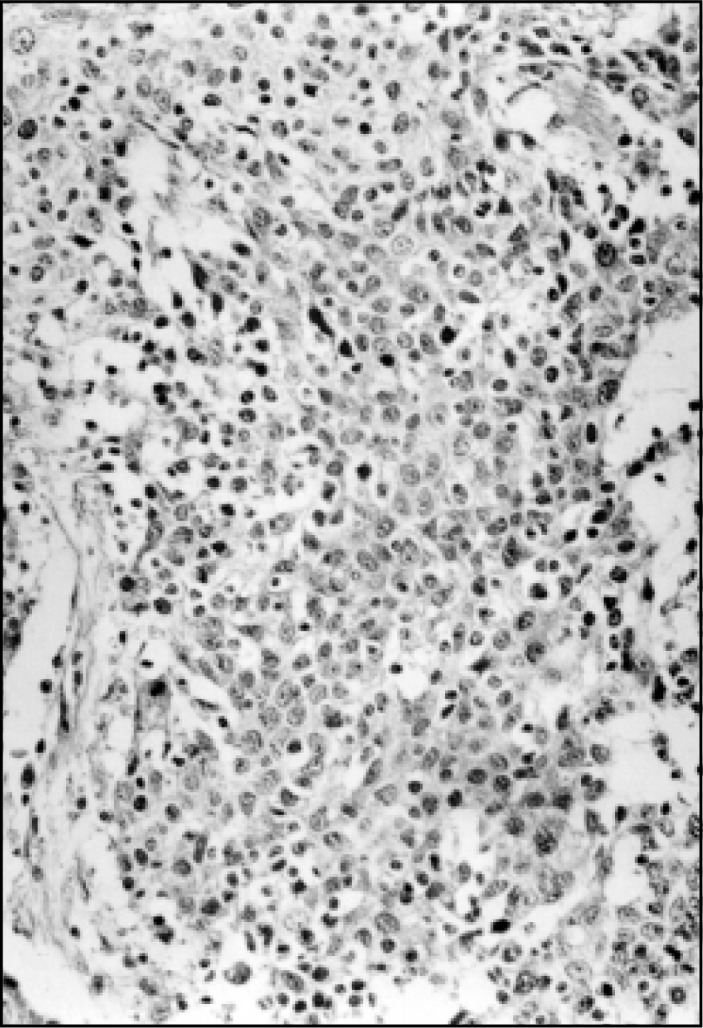
High malignancy grade MEC of a salivary gland. Note the proliferation of epidermoid cells showing hyperchromatic or vesicular pleomorphic nuclei, changes in the nuclear/cytoplasmic relationship, some mitotic figures and a few mucin cells. (Stained with hematoxylin and eosin with magnification of 400x).

**Figure 4:**
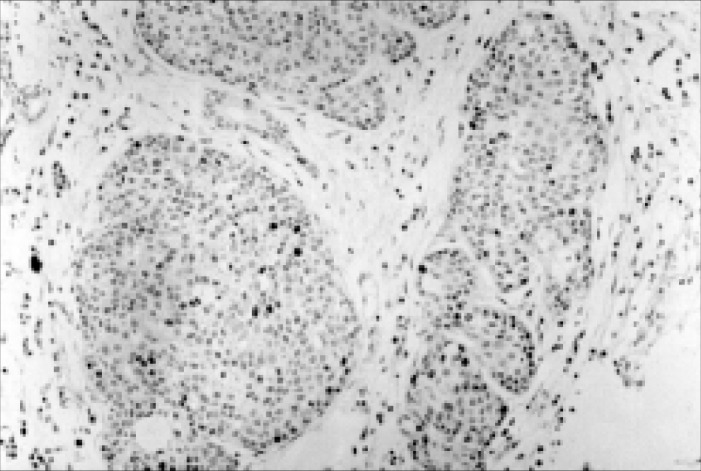
Immunohistochemical studies of MEC of a salivary gland, with a moderate number of cells positive for PCNA antigen expression. The tumor cells were considered positive when they showed a brownish stain. (250x magnification).

- *Conventional histopathological study.* For this analysis, tissue sections with 3-4 mm thickness, fixed in 10% formaldehyde solution and embedded in paraffin were examined. The slides were stained with hematoxylin and eosin and studied using optical microscopy. The tumors were graded for malignancy, according to the criteria of Seifert & Sabin^5,6^ and Auclair, Goode & Ellis,^17^ as follows: (a) Low grade: highly differentiated neoplasia with predominance of macro and microcysts. Presence of intermediate and mucin-producing cells (*Figure 1*); (b) Intermediate grade: predominance of intermediate cells and few cysts. Presence of mucin-producing cells and islands of epidermoid cells (*Figure 2*); (c) High grade: poorly differentiated neoplasia with predominance of intermediate and epidermoid cells in solid blocks. Mucin-producing cells are present in less than 10% (*Figure 3*).
- *Immunohistochemical study for PCNA determination.* For immunohistochemical PCNA detection, the streptavidin biotin technique was used.^15^ The monoclonal antibody used was PC10 (DAKO Corporation, Glostrup, Denmark) in a 1/100 dilution with 18 hours incubation time. Tumor cells that showed a brownish stain were considered positive (*Figure 4*). The quantitative expression of PCNA was obtained from the relationship between the number of PCNA-positive cells and the total number of evaluated cells in percentage terms. The total number of evaluated cells was never less than 1000 cells in each procedure. Two evaluations were performed for each tumor. In all cases the immunohistochemical evaluation of PCNA was performed by the pathologist in a blind manner (without knowing the histopatho-logical diagnosis or the malignancy grade).

### Main Measurement

The correlation between PCNA expression and the histological grade of malignancy in MEC of salivary glands.

### Statistical methods

The statistical analysis was performed using the SAS/STAT program, users’ guide version 6.0 (SAS Institute Inc., 1989), to organize the prediction interval for the three grades of malignancy. Student's t distribution test and Tukey's multiple comparison test were used to evaluate the differences between the obtained data.

## RESULTS

### Clinical data

The clinical data of the 11 patients are shown in Table 1. The median age was 45.7 years (range 36-75). Nine patients were white, 1 black and 1 unknown. The male to female proportion was approximately 1:1 (6 male and 5 female). The primary tumor sites were: parotid gland (64%), hard palate, base of tongue, submaxillary gland and mouth floor (9% each). The mean duration of complaints was 5.6 years and the mean tumor size was 5.3 cm. Seven patients were asymptomatic and only 4 presented symptoms at the time of diagnosis.

**Table 1:** Clinical data on 11 cases of salivary gland mucoepidermoid carcinoma - Head and Neck Surgery Service - Heliópolis Hospital, São Paulo - 1978 to 1997

Case N°	Age (year)	Gender	Race	Symptoms	Location	Tumor Size (cm)	Duration of Complaints
1	26	F	White	Asymptomatic	Parotid	3.0	14 y
2	75	F	White	Symptomatic	Parotid	7.0	5 y
3	73	M	White	Asymptomatic	Submandibular	5.0	1 y
4	44	M	White	Asymptomatic	Parotid	10.0	1 y
5	46	F	White	Asymptomatic	Base of tongue	5.0	na*
6	51	M	Black	Symptomatic	Floor of mouth	2.5	14 y
7	36	F	White	Asymptomatic	Parotid	6.0	5 y
8	43	M	White	Symptomatic	Hard palate	2.5	15 y
9	na*	M	na*	na*	Parotid	6.0	3 y
10	52	F	White	Symptomatic	Parotid	3.0	1 y
11	57	M	White	Asymptomatic	Parotid	12.0	3 y

* na = not available.

### Histopathological and immunohistochemical findings

The results of histopathological evaluation showed 4 cases (36%) classified as low grade, 4 cases (36%) intermediate and 3 cases (27%) as high malignancy grade. Table 2 presents the relative and absolute values of PCNA-positive cells in the two analyses performed.

**Table 2:** Comparison between the histopathological classification and relative and absolute values of proliferating cell nuclear antigen (PCNA+) cells for all cases

Case N°	Histopathological Classification	1st Evaluation			2nd Evaluation		
			PCNA+ cells			PCNA+ cells	
		Total cells	Absolute	Relative	Total cells	Absolute	Relative
1	Low grade	1268	196	15.5%	1186	172	14.5%
3	Low grade	907	141	15.5%	1109	151	13.6%
8	Low grade	1069	213	19.9%	1116	179	16.0%
10	Low grade	1120	210	18.8%	1113	161	14.5%
2	Intermediate grade	1180	247	21.0%	1122	253	22.6%
4	Intermediate grade	1842	375	20.4%	1109	272	24.5%
6	Intermediate grade	1231	462	37.5%	1324	492	37.2%
7	Intermediate grade	1508	535	35.5%	1761	683	38.8%
5	High grade	1217	437	35.9%	1224	537	43.9%
9	High grade	1146	726	63.4%	1206	921	68.1%
11	High grade	1117	742	66.4%	1079	693	64.2%

When the mean values of positive expression of PCNA in the different groups of histological classification were analyzed, a tendency to increase this expression was observed in cases with a high malignancy grade, as shown in Table 3. The variance analysis using Tukey's test showed significant differences between the mean value of the high-grade malignancy group when compared with the low and intermediate grade groups (P<0.01). These latter groups did not present differences between each other.

**Table 3:** Mean percentage and dispersion values of proliferating cell nuclear antigen (PCNA+) cells for all patients and in each group according to the histopathological classification

Histopathological Classification Malignancy grade	Statistical parameters	Relative values of PCNA+ cells
All groups	n Mean	11 (18.9) 32.2%
Low grade group	n Mean	4 (1.6) 16.0% *
Intermediate grade group	n Mean	4 (8.7) 29.7% *
High grade group	n Mean	3 (14.8) 56.9% **

* not significant statistical difference;

** significant statistical difference (P<0.01); Standard Deviation given in parenthesis.

## DISCUSSION

Although MEC was defined as a specific entity five decades ago, controversies persist concerning the histopathological malignancy grades, survival and prognosis in these tumors.^18,19^

A comparison of the results of this study with data in the literature showed agreement regarding the most frequent site (parotid gland in 64%),^2,20^ age (fifth decade)^20^ and a larger prevalence in white individuals.^2, 21, 22^ The time taken for the disease to evolve (5.3 years) was similar to that observed by Auclair & Ellis^2^ and Loyola et al (6.2 years). In spite of disagreement with some reports in the literature indicating female predominance,^22–24^ the similarity in proportions between the sexes that we found concords with other descriptions in which equal proportions were encountered.^4,20, 25^

It is widely accepted that the histological malignancy grade of MEC is related to a more unfavorable outcome in high grade tumors when compared with other grades.^2, 6,19,26^ There is, however, a large variation in pathological criteria used in classifying these tumors, while the evaluation of the tumor proportion of cystic areas forms part of all current grading systems. In many cases there is difficulty in precisely determining the cystic component. Additional classification criteria may be useful under these circumstances.

Data presented here show that the evaluation of cell proliferation using PCNA has a close relationship to malignancy grades determined by histopathological methods.

Contrary to the results obtained by Tsai & Jin,^27^ the data of the present study showed a statistically significant difference between the malignancy grades and the expression of PCNA in tumor cells, as evaluated by the percentage of positive cells. Tumors with a high grade of malignancy showed a greater percentage of PCNA-positive cells than the tumors with intermediate or low grade.

These findings suggest that the evaluation of PCNA expression in MEC of salivary glands can be used as a complementary procedure for appropriate classification of these tumors. Therefore, more studies are necessary to determine the role of PCNA positivity in treatment and prognosis of these tumors.

## References

[1] Thackray AC, Lucas RB (1974). Tumors of the major salivary glands. Atlas of Tumor Pathology, 2nd Series, Fascicle 10.

[2] Auclair PL, Ellis GL, Ellis GL, Auclair PL, Gneep DR (1991). Mucoepidermoid carcinoma.

[3] Franzi SA, Carvalho MB (1997). Carcinoma mucoepidermóide avançado das glândulas salivares. Rev Bras Cancerol.

[4] Loyola AM, Araújo VC, Sousa SOM, Araújo NS (1995). Minor salivary glands tumors. A retrospective study of 164 cases in a Brazilian population. Oral Oncol Eur J Cancer.

[5] Seifert G, Sobin LH (1991). World Health Organization histological classification of tumors. Histological typing of salivary gland tumors.

[6] Seifert G, Sobin LH (1992). The World Health Organization Histological classification of salivary gland tumors: a commentary on the second edition. Cancer.

[7] Spiro RH, Huvos AG, Berk R, Strong EW (1978). Mucoepidermoid carcinoma of salivary gland origin. Am J Surg.

[8] Rapoport A, Carvalho MB, Fava AS (1988). Diagnóstico e tratamento das neoplasias das glândulas salivares menores: estudo de 55 casos. Rev Col Bras Cirur.

[9] Ogawa I, Miyachi M, Takata T, Vuhahula E, Ijuhin N, Nikai H (1993). Proliferative activity of salivary gland pleomorphic adenoma and myoepithelioma as evaluated by the proliferating cell nuclear antigen (PCNA) labeling index (LI). J Oral Pathol Med.

[10] Tsuji T, Sasaki K, Kimura Y, Yamada K, Mori M, Shinozaki F (1992). Measurement of proliferating cell nuclear antigen (PCNA) and its clinical application in oral cancers. lnt J Oral Maxillofac Surg.

[11] Miyachi K, Fritzler MJ, Tan EM (1978). Autoantibody to a nuclear antigen in proliferating cells. J Immunol.

[12] Bravo R, Frank R, Blundell PA, MacDonald-Bravo H (1987). Cyclin/PCNA is the auxiliary protein of DNA polymerase d. Nature.

[13] McCormick D, Hall PA (1992). The complexities of proliferating cell nuclear antigen. Histopathology.

[14] Garcia RL, Araújo VC, Araújo NS (1993). Argyrophilia in nucleolar organizer regions (AgNOR) in adenoid cystic carcinoma and polymorphous low-grade adenocarcinoma of the salivary glands. Eur Arch Otorhinolaryngol.

[15] Hall PA, Levinson DA, Woods AAL (1990). Proliferating cell nuclear antigen (PCNA) immunolocalization in paraffin expression: an index of cell proliferation with evidence of deregulated expression in some neoplasms J Pathol.

[16] Kurki P, Vanderlaan M, Dolbear F, Gray J, Tam EM (1986). Expression of proliferating cell nuclear (PCNA) cyclin during cell cycle. Exp Cell Res.

[17] Auclair PL, Goode RK, Ellis GL (1992). Mucoepidermoid carcinoma of intraoral salivary glands: evaluation and application of grading criteria in 143 cases. Cancer.

[18] Eversole LR (1970). Mucoepidermoid carcinoma: a review of 815 cases. J Oral Surg.

[19] Hicks MJ, El-Naggar AK, Flaitz CM, Luna MA, Batsakis JG (1995). Histocytological grading of mucoepidermoid carcinoma of major salivary glands in prognosis and survival: a clinicopathological and flow cytometric investigation. Head & Neck.

[20] Eveson JW, Cawson RA (1958). Tumors of the minor (oropharyngeal) salivary glands: a demographic study of 336 cases. J Oral Pathol.

[21] Evans RW, Cruickshank AH, Evans RW, Cruickshank AH (1970). Mucoepidermoid tumors. Epithelial tumors of the salivary glands.

[22] Eversole LR, Rivin S, Sabes WR (1972). Mucoepidermoid carcinoma of minor salivary glands: report of 17 cases with follow-up. J Oral Surg.

[23] Batsakis JG (1979). Tumors of the Head and Neck.

[24] Eneroth CM, Hjertman L, Moberger G (1970). Mucoepidermoid carcinoma of the palate. Acta Otolaryng.

[25] Goode RK, Auclair PL, Ellis GL (1998). Mucoepidermoid carcinoma of the major salivary glands. Clinical and histopathological analysis of 234 cases with evaluation of grading criteria.

[26] Healey WV, Perzin KH, Smith L (1970). Mucoepidermoid carcinoma of salivary gland origin. Classification clinical correlation and results of treatment. Cancer.

[27] Tsai ST, Jin YT (1995). Proliferating cell nuclear antigen (PCNA) expression in oral squamous cell carcinoma. J Oral Pathol Med.

